# Implementation of the Rauch-Tung-Striebel Smoother for Sensor Compatibility Correction of a Fixed-Wing Unmanned Air Vehicle

**DOI:** 10.3390/s110403738

**Published:** 2011-03-28

**Authors:** Woei-Leong Chan, Fei-Bin Hsiao

**Affiliations:** Institute of Aeronautics and Astronautics, National Cheng Kung University, Tainan City 701, Taiwan; E-Mail: fbhsiao@mail.ncku.edu.tw

**Keywords:** Unmanned Air Vehicle (UAV), sensor data compatibility, Rauch-Tung-Striebel (RTS), Wiener type filter, noise estimation

## Abstract

This paper presents a complete procedure for sensor compatibility correction of a fixed-wing Unmanned Air Vehicle (UAV). The sensors consist of a differential air pressure transducer for airspeed measurement, two airdata vanes installed on an airdata probe for angle of attack (AoA) and angle of sideslip (AoS) measurement, and an Attitude and Heading Reference System (AHRS) that provides attitude angles, angular rates, and acceleration. The procedure is mainly based on a two pass algorithm called the Rauch-Tung-Striebel (RTS) smoother, which consists of a forward pass Extended Kalman Filter (EKF) and a backward recursion smoother. On top of that, this paper proposes the implementation of the Wiener Type Filter prior to the RTS in order to avoid the complicated process noise covariance matrix estimation. Furthermore, an easy to implement airdata measurement noise variance estimation method is introduced. The method estimates the airdata and subsequently the noise variances using the ground speed and ascent rate provided by the Global Positioning System (GPS). It incorporates the idea of data regionality by assuming that some sort of statistical relation exists between nearby data points. Root mean square deviation (RMSD) is being employed to justify the sensor compatibility. The result shows that the presented procedure is easy to implement and it improves the UAV sensor data compatibility significantly.

## 1. Introduction

Air vehicle sensor compatibility is a specific field of study dedicated to the estimation of onboard sensor errors. The sensor errors are usually described as the scale factors and the biases. The field is often discussed in the context of flight path reconstruction. The term “compatibility” refers to the consistency of the measured data with the force equations that govern the airdata (airspeed, AoA, and AoS), and the kinematic equations that govern the attitude angles (roll, pitch, yaw). If flight path reconstruction is to be discussed jointly, the navigational equations will be included. Sensor compatibility is often the first step of the so-called “two-step method” of aircraft system identification. The first step is the sensor data correction/estimation; the second part is the parameter estimation.

Research on the topic of aircraft sensor compatibility started as early as the 1970s. In 1976, Jonker [1] pioneered the research in the area by investigating the applicability of the Kalman Filter for flight path reconstruction and estimation of instrumentation biases. A year later, the National Aeronautics and Space Administration (NASA) published a report on the measured aircraft responses compatibility check. As discussed in the report, Klien and Schiess [2] implemented the EKF with a fixed-point smoother to accomplish the task. In 1984, similar work was again published by NASA. This time, Whitmore *et al.* [3] calibrated the airdata error using the Linearized Kalman Filter (LKF). Up to that point, The Kalman Filter and EKF were proven to be sufficient in estimating the air vehicle sensor errors. However, the implementations of Kalman Filter and EKF are not easy without *a priori* knowledge of the measurement noise and process noise and this noise information is often not easily available. In response to that issue, Chu *et al.* [4] proposed the implementation of the modified recursive Maximum Likelihood adaptive filter in 1995, claiming that the adaptive filter is more robust than the EKF. Mendonça *et al.* [5] also proposed an adaptive method for estimating the noise properties in 2007. All these efforts were carried out using manned aircraft.

In the age of the UAV, due to the advances in discrete time system identification techniques and control theories, identifying an accurate model that is coherent with the force equations and kinematic equations seems to be unnecessary. However, discrete time system identification techniques are unable to provide a good insight of an aircraft’s dynamics because the discrete time models are usually physically meaningless, no matter how well they agree with the real flight data. In addition to that, UAVs are usually smaller than manned aircrafts, and fly at lower velocities, and thus are very sensitive to the atmospheric turbulence which will result in a higher noise ratio in the flight data. Due to that, sensor compatibility correction is more important than ever if the dynamic characteristics of an UAV are to be investigated.

Motivated by the discussion above, this paper attempts to derive a procedure in order to perform compatibility correction on the flight data of the SP-80 UAV. The SP-80 UAV [6–9] is an UAV research testbed developed and operated under the Spoonbill UAV Project by the Remotely Piloted Vehicle and Micro-Satellite Laboratory (RMRL) of the National Cheng Kung University (NCKU), Taiwan. The Spoonbill UAV Project was established to fulfill educational research purposes of which it has brought forth the successful implementation of subspace and prediction-error method identification algorithms in the UAV system identification [7], implementation of the Linear Quadratic Gaussian (LQG) controller to the autopilot system [8] and a successful cross-sea automatic flight demonstration that covered 90.2 km [6]. As part of the continuing researches on the SP-80 UAV, the work presented in this paper intends to bridge the gap between the controller implementation and the understanding of the dynamics of the UAV.

The following sections in this paper are organized as follows: first, Section 2 introduces the Spoonbill UAV system. In particular, the sensor system is described in detail. Section 3 reviews the force equations and kinematic equations. This is followed by Sections 4 and 5 which briefly elaborate the Wiener Type Filter and the RTS smoother. Section 6 presents the flight test data and initial data reconstruction and the next section explains the sensor compatibility correction procedure in detail. Then, the result of correction is discussed in Section 8. Finally, Section 9 draws the conclusions for the paper.

## 2. The Hardware Architecture

### 2.1. The Spoonbill UAV System

**Figure 1.**
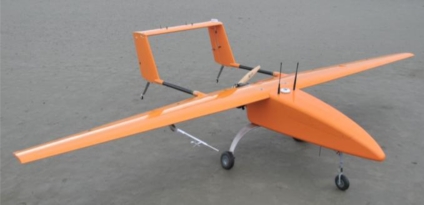
The SP-80 UAV.

The Spoonbill UAV system used in this work is designed and operated by the RMRL for educational research purposes. The air vehicle used is designated as the SP-80, which has a high-wing, twin-boom, pusher engine configuration and the power plant is an 80 c.c. two stroke gasoline engine. Figure 1 depicts the air vehicle. The 3.50 m wingspan air vehicle is capable of carrying 3 kg of fuel and it has a maximum endurance of an hour once fully refueled. The fuselage is designed such that it can carry various sensors, an onboard computer, telemetry devices, and a gimbaled camera system. So far, the air vehicle is capable of performing automatic flight using a flight controller based on the LQG algorithm, and it automatically flew the longest distance of 90.2 km on 20 October 2009 [6].

### 2.2. The Onboard System

**Figure 2.**
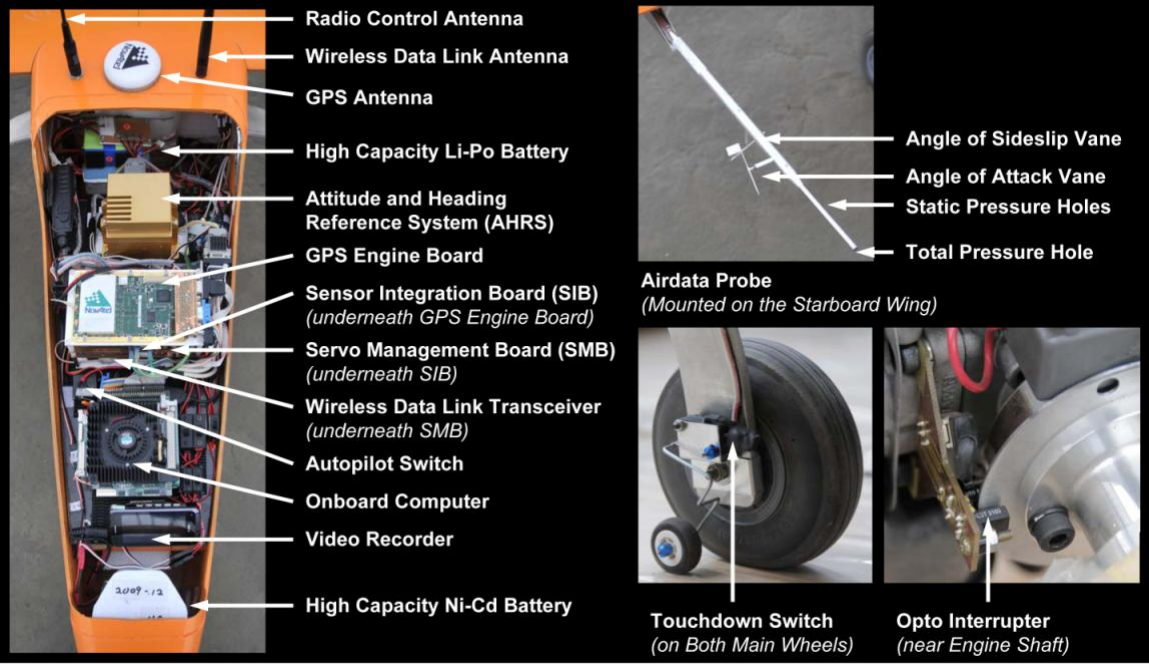
The onboard system.

**Figure 3.**
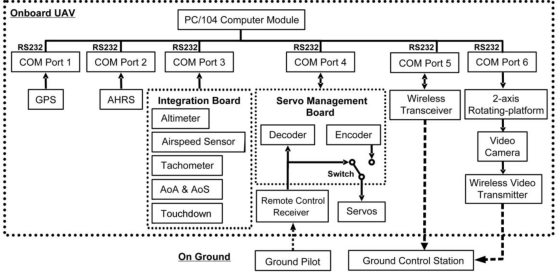
Spoonbill UAV System architecture.

Figure 2 shows the onboard avionics system and Figure 3 illustrates the Spoonbill UAV system architecture. The onboard computer acts as the central processing unit. It is a PC/104 embedded computer produced by Advantech Co., Ltd. The onboard computer is operating under a Windows-XP embedded operating system and the flight control computer program is written using Borland C++ Builder. The onboard computer collects data from the peripheral sensors through RS-232 protocol via several communication ports (COM Port). In addition to that, the onboard computer also performs bi-directional communication with the Servo Management Board (SMB), and the wireless transceiver. The final COM Port is occupied by the rotation platform of the gimbaled camera system. The flight control program operates under various modes depends on the system operator and the flight test objectives. The basic functions of the flight control program are: acquires data from the peripheral sensors, executes the automatic flight control algorithm, and records selected flight data. All of these operations are executed at the rate of 20 Hz.

Before any discussion on the peripheral sensors can commence, it is worth mentioning that the GPS receiver, the AHRS, the wireless transceiver, and the video camera system are all off-the-shelf products, while the Sensor Integration Board (SIB), and the SMB were designed and fabricated in-house by the RMRL. The SMB is a crucial component of an autopilot system for it decodes the control signal from the pilot in Pulse Width Modulation (PWM) form during the manual flight mode, and it encodes PWM signal to control the air vehicle control surface actuators (servo motors) according to the command given by the onboard computer. The SMB is capable of decoding and encoding nine channels of PWM signals at a 20 Hz update rate. The pilot can easily cede flight control authority to the onboard computer by flipping a switch on the remote control radio. Regaining control of the air vehicle from the onboard computer is just as easy.

**Figure 4.**
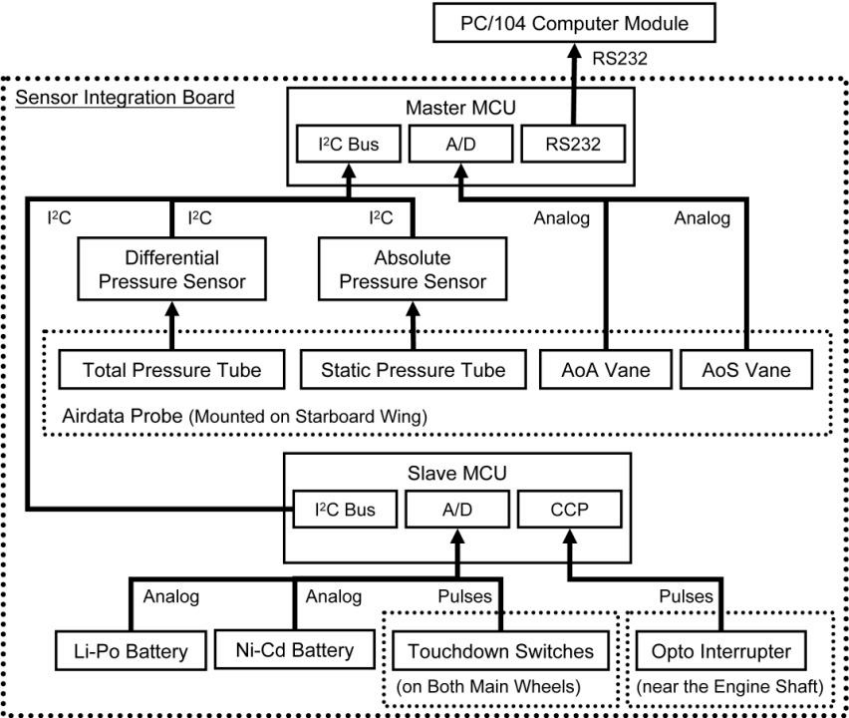
Sensor Integration Board architecture.

The peripheral sensors are divided into three major groups. The first group is the GPS. The GPS receiver is the Novatel OEMV-3. Unlike any other GPS receivers built for automobile navigation that normally operating at a maximum of 5 Hz, The Novatel OEM-V3 is capable of providing global position and ground speed at the rate of 20 Hz and is thus consistent with the sampling rate of other onboard sensors. The second group is the AHRS. It is a Crossbow AHRS440. The AHRS measures 3-axis accelerations, angular rates, and attitude angles. The attitude angle measurement is augmented by GPS information from the Novatel OEM-V3. The final group is the SIB. The SIB integrates all other sensors using microcontroller units (MCU). It is designed and fabricated in-house by the RMRL. The MCUs on the SIB are responsible to collect data from the corresponding sensors, perform signal conditioning on the data (such as data filtering), and pass the data to the onboard computer via RS232 protocol. Sensors included in the SIB are the differential air pressure sensor for airspeed measurement, absolute air pressure sensor for barometric altitude measurement, engine speed tachometer, AoA and AoS, and the touchdown sensors. Figure 4 illustrates the SIB architecture.

The SIB consists of two MCUs: the Master MCU and the Slave MCU. The Master MCU is the core of the SIB. It controls the data flow within the SIB and sends data to the onboard computer. There are two sensors built on the SIB: the SenSpecial SCPB-MB0/10D100i2c32667R5 digital differential pressure sensor, and the SenSpecial SCPB-mmHg525/825A100i2c32667R5 digital absolute pressure sensor. Both pressure sensors are connected to the air pressure tubes from the SpaceAge Control 101100-02 airdata probe. The airdata probe is mounted on the starboard wing, as shown in Figure 2. The Master MCU communicates with the pressure sensors via I^2^C protocol, and calculates the airspeed and barometric altitude using the pressure readings and the following equations.

(1) $$h=h_{b}+\frac{T_{b}}{L_{b}}\left[{\left(\frac{P}{P_{b}}\right)}^{-\frac{R^{*}L_{b}}{g_{0}M}}-1\right]$$

(2) $$V=\sqrt{\frac{2\left(P_{0}-P\right)}{\rho }}$$

(3) $$\rho =\frac{P}{\mathrm{RT}}$$

(4) $$T=T_{b}+L_{b}h$$

Where

*g*_0_ = 9.80665*m*/*s*^2^ is the gravitational acceleration at sea level
*h* is the geopotential altitude in meter
*h_b_* = 0 is the sea level altitude
*L_b_* = −6.5×10^−3^ *K/m* is the temperature lapse rate from sea level to altitude 11,000 m
*M* = 0.0289644 *kg/mol* is the molar mass of Earth’s air
*P* is the static pressure in Newton per square meter (Pascal)
*P*_0_ is the total pressure in Newton per square meter (Pascal)
*P_b_* = 1.01325×10^5^ *N/m*^2^ is the sea level static pressure
*R* = 287 *J*/(*kg* · *K*) is the specific gas constant
R^*^ = 8.31432*N* · *m*/(*mol · K*) is the universal gas constant
*T* is the temperature
*T_b_* = 288.16*K* is the sea level temperature
*V* is the airspeed in meter per second
*ρ* is the air density in kilogram per cubic meter

Equation 1 is called the barometric formula introduced by NASA in 1976 [10]. It has since become the standard equation for barometric altitude calculation. Equation 2 is derived from the Bernoulli’s equation [11]. From Equation 2, the airspeed of the air vehicle can be determined once the total pressure, static pressure and air density are known. Due to the absence of an air density sensor, the air density is estimated using Equation 3. The temperature needed in Equation 3 is calculated by one of the standard atmosphere formulae shown here as Equation 4 [10]. Equation 4 is only valid from sea level to altitude 11,000 m. The absolute pressure sensor measures *P*, while the differential pressure sensor measures (*P*_0_ − *P*).

The airdata probe is also built with two vanes, one for the AoA measurement, and another for the AoS. The vanes are basically two delicate potentiometers. The SIB provides the operating voltage to the vanes and senses the vanes’ movement through the Master MCU 12 bit analog-to-digital converters (A/D).

The onboard avionics are powered by a high capacity Lithium-Polymer (Li-Po) battery and the air vehicle control surface actuators (servo motors) are powered by high capacity Nickel-Cadmium (Ni-Cd) batteries. The Slave MCU reads the battery voltages via a built-in 10 bit A/D. In addition, it reads the pulse signal from the touchdown switches mounted on both main wheels of the air vehicle; such to determine the instant the air vehicle lifts off the ground and touches down. The most important function of the Slave MCU is to calculate the engine rotation speed. The opto interrupter mounted near the engine shaft generates a pulse per engine revolution. The Capture/Compare/PWM (CCP) interrupt of the Slave MCU detects the pulse and calculates the time taken between two pulses before converts it to revolution per minute (rpm). The Slave MCU sends the data string to the Master MCU upon Master MCU’s I^2^C request.

## 3. Equations of Motions

The air vehicle translation and rotation dynamics are governed by two sets of ordinary differential equations (ODE), namely the force equations and the kinematic equations. Each set of these equations consists of three equations, and each equation governs the motion of one axis. The detailed derivation of the equations has been described well by Nelson [12].

### 3.1. Force Equations

The force equations as shown in Equation 5 describe the variation of the three body axis velocities. The ODEs are functions of the velocities, accelerations, angular rates, and gravity components:

(5a) $$\dot{u}=\mathrm{rv}-\mathrm{qw}+a_{x}-g\;\text{sin}\;\theta$$

(5b) $$\dot{v}=\mathrm{pw}-\mathrm{ru}+a_{y}+g\;\text{cos}\;\theta \;\text{sin}\;\phi$$

(5c) $$\dot{w}=\mathrm{qu}-\mathrm{pv}+a_{z}+g\;\text{cos}\;\theta \;\text{cos}\;\phi$$

where:

*a_x_* is the acceleration along the body X-axis
*a_y_* is the acceleration along the body Y-axis
*a_z_* is the acceleration along the body Z-axis
*g* = 9.81*ms*^−2^ is the gravitational acceleration
*p* is the roll rate
*q* is the pitch rate
*r* is the yaw rate
*u* is the velocity along the body X-axis
*v* is the velocity along the body Y-axis
*w* is the velocity along the body Z-axis

It is also possible to describe the force equation in terms of the variation of airspeed, AoA, and AoS. The airspeed, AoA, and AoS are related to the body axis velocities as in Equation 6. Thus, the force equations can be transformed into Equation 7 that describe the variation of airspeed, AoA, and AoS:

(6a) $$V=\sqrt{u^{2}+v^{2}+w^{2}}$$

(6b) $$\alpha ={\text{tan}}^{-1}\left(\frac{w}{u}\right)$$

(6c) $$\beta ={\text{sin}}^{-1}\left(\frac{v}{V}\right)$$

(7a) $$\begin{array}{ll} \dot{V}= & \left(a_{x}\;\text{cos}\;\alpha +a_{z}\;\text{sin}\;\alpha \right)\;\text{cos}\;\beta +a_{y}\;\text{sin}\;\beta +\\ & g\left(\text{cos}\;\theta \;\text{cos}\;\phi \;\text{sin}\;\alpha \;\text{cos}\;\beta +\text{cos}\;\theta \;\text{sin}\;\phi \;\text{sin}\;\beta -\text{sin}\;\theta \;\text{cos}\;\alpha \;\text{cos}\;\beta \right) \end{array}$$

(7b) $$\begin{array}{ll} \dot{\alpha }= & \frac{1}{V\;\text{cos}\;\beta }\left\{a_{z}\;\text{cos}\;\alpha -a_{x}\;\text{sin}\;\alpha +g\;\left(\text{cos}\;\theta \;\text{cos}\;\phi \;\text{cos}\;\alpha +\text{sin}\;\theta \;\text{sin}\;\alpha \right)\right\}+\\ & q-\text{tan}\;\beta \left(p\;\text{cos}\;\alpha +r\;\text{sin}\;\alpha \right) \end{array}$$

(7c) $$\dot{\beta }=\frac{1}{V}\left\{\begin{array}{l} a_{y}\;\text{cos}\;\beta -\left(a_{x}\;\text{cos}\;\alpha +a_{z}\;\text{sin}\;\alpha \right)\;\text{sin}\;\beta +\\ g\left[\text{cos}\;\theta \;\text{sin}\;\phi \;\text{cos}\;\beta +\left(\text{sin}\;\theta \;\text{cos}\;\alpha -\;\text{cos}\;\theta \;\text{cos}\;\phi \;\text{sin}\;\alpha \right)\text{sin}\;\beta \right] \end{array}\right\}+p\;\text{sin}\;\alpha -r\;\text{cos}\;\alpha$$

where:

*V* is the airspeed
*α* is the AoA
*β* is the AoS

As a matter of fact, even though Equation 7 is much more complicated compared to Equation 5, it is more intuitive to use Equation 7 rather than Equation 5 because the airspeed, AoA, and AoS are normally measured by the onboard instruments instead of using the body axis velocities.

### 3.2. Kinematic Equations

The kinematic equations as shown in Equation 8 describe the variation of the three attitude angles, namely the roll angle, pitch angle, and yaw angle. The ODEs are functions of the attitude angles, and angular rates:

(8a) $$\dot{\phi }=p+\text{tan}\;\theta \left(q\;\text{sin}\;\phi +r\;\text{cos}\;\phi \right)$$

(8b) $$\dot{\theta }=q\;\text{cos}\;\phi -r\;\text{sin}\;\phi$$

(8c) $$\dot{\psi }=\frac{q\;\text{sin}\;\phi +r\;\text{cos}\;\phi }{\;\text{cos}\;\theta }$$

where:

*ϕ* is the roll angle
*θ* is the pitch angle
*ϕ* is the yaw angle

## 4. Wiener Type Filter

The Wiener Type Filter [13] is an optimal filter in the frequency domain. Equation 9(a) indicates that the measurement is a summation of the filtered/true signal and noise. In order to simplify the transformation to the frequency domain, the measurement is modified using Equation 9(b). Equation 9(b) is an odd function with its end point discontinuity removed. Such function allows the Fourier sine series expansion. The Fourier sine series coefficients are given by Equation 9(c):

(9a) $$z_{k}=y_{k}+n_{k},\;\;k=1,2,\ldots ,N$$

(9b) $$g_{k}=z_{k}+z_{1}-\left(k-1\right)\left[\frac{z_{N}+z_{1}}{N-1}\right],\;k=1,2,\ldots ,N$$

(9c) $$b_{l}=\frac{2}{N-1}\sum_{l=2}^{N-1}g_{l}\;\text{sin}\;\left[\frac{l\pi \left(k-1\right)}{N-1}\right],\;i=1,2,\ldots ,N-1$$

where:

*b_l_* is the Fourier sine series coefficient at *l*^th^ frequency index
*g_k_* is the modified measurement at *k*^th^ time step
*N* is the data size
*n_k_* is the noise at *k*^th^ time step
*y_k_* is the filtered/true at *k*^th^ time step
*z_k_* is the measurement at *k*^th^ time step

The next step is to define the true signal model and noise model. It is important to know that the filter consists of weighting indices range from 0 to 1, where 0 indicates total rejection and 1 indicates otherwise. Thus, it is ideal when the filter equals to 0.5 as the frequency index then corresponds to the cutoff frequency. Plus, as discussed by Morelli, it is best to describe the true signal model being proportional to *l*^−3^. Given the form of the Wiener Type Filter as in Equation 10(a), yields the true signal model and noise model as shown in Equation 10(b,c). Equation 10(d) shows how the frequency index being related to the frequency. Similarly, Equation 10(e) determines the frequency index corresponds to the desired cutoff frequency. The filter is applied in Equation 10(f) to obtain the filtered modified measurement. Finally, Equation 10(g) reconstructs the filtered measurement:

(10a) $$\Phi_{l}=\frac{Y_{l}^{2}}{Y_{l}^{2}+N_{l}^{2}},\;\;0\leq l\leq N-1$$

(10b) $$Y_{l}=l_{c}{}^{3}l^{-3}$$

(10c) $$N_{l}=1$$

(10d) $$f_{l}=\frac{l}{2\left(N-1\right)\Delta t}$$

(10e) $$l_{c}=2\;f_{l}\;\left(N-1\right)\Delta t,\;\;\text{if}\;\;f_{l}\leq f_{c,d}<f_{l+1}$$

(10f) $${\tilde{g}}_{s,k}=\sum_{l=1}^{N-1}\Phi_{l}b_{l}\;\text{sin}\;\left[\frac{l\pi \left(k-1\right)}{N-1}\right],\;k=1,2,\ldots ,N$$

(10g) $$y_{k}={\tilde{g}}_{s,k}+z_{1}+\left(k-1\right)\frac{z_{N}-z_{1}}{N-1},\;\;k=1,2,\ldots ,N$$

where:

*f_l_* is the frequency at *l*^th^ frequency index
*f*_c,d_ is the desired cutoff frequency
*g̃*_s,*k*_ is the filtered modified measurement at *k*^th^ time step
*l*_c_ is the frequency index corresponds to the cutoff frequency
*N_l_* is the noise model in discrete frequency domain at *l*^th^ frequency index
*Y_l_* is the true signal model in discrete frequency domain at *l*^th^ frequency index
**Φ***_l_* is the filter at *l*^th^ frequency index

## 5. Rauch-Tung-Striebel Smoother

The RTS Smoother [14–16] is a two pass algorithm. The forward pass is a standard EKF [15] while the backward recursion is introduced to reduce the inherent bias in the EKF estimates.

### 5.1. Forward Pass (Extended Kalman Filter)

The EKF is literally one of the most widely used state estimation methods due to its simplicity. The EKF is derived from the standard Kalman Filter. The Kalman Filter is derived based on linear systems, but the EKF works for nonlinear systems by performing linearization about the current estimates.

Over the years, different forms of EKF have been proposed, namely the continuous time EKF, discrete time EKF, and the continuous-discrete EKF. Since the force equations and kinematic equations are continuous time equations and the flight test measurements are discrete, the continuous-discrete EKF is used for the work presented in this paper. The continuous-discrete EKF is presented here as Equations 11–12. Often, the difficulty of implementing the EKF is mainly due to the lack of *a priori* knowledge of the noise covariance.

Given a nonlinear continuous time dynamic system with discrete time measurement, of which the measurement and process noise are Gaussian distributed:

(11a) $$\dot{\text{x}}(t)=f\left(\text{x}(t),\;\text{u}\;(t)\right)+\text{w}\;(t),\;\text{w}(t)∼N\left(0,\text{Q}(t)\right)$$

(11b) $${\text{z}}_{k}=h\left({\text{x}}_{k}\right)+{\text{v}}_{k},\;\;{\text{v}}_{k}∼N\left(0,{\text{R}}_{k}\right)$$

where:

*f*(**x**(*t*),**u**(*t*)) is the nonlinear ordinary differential equations
*h*(**x***_k_*) is the measurement equations
*k* is the number of time step
**Q** is the process noise covariance matrix
**R** is the measurement noise covariance matrix
**u** is the input vector
**v** is the Gaussian distributed measurement noise vector
**w** is the Gaussian distributed process noise vector
**x** is the state vector
**z** is the measurement vector

The prediction phase:

(12a) $$\hat{\text{x}}\left(t\right)=\int_{t}^{t+\Delta t}f\left(\hat{\text{x}}\left(t\right),\text{u}\left(t\right)\right)dt,\;\;\text{F}\left(t\right)={\frac{\partial f}{\partial \text{x}}|}_{\hat{\text{x}}\left(t\right),\text{u}\left(t\right)}$$

(12b) $$\dot{\text{P}}\left(t\right)=\text{F}\left(t\right)\text{P}\left(t\right)+\text{P}\left(t\right)\text{F}{\left(t\right)}^{T}+\text{Q}\left(t\right)$$

(12c) $${\hat{\text{z}}}_{k}=\;\;h\left({\hat{\text{x}}}_{k|k-1}\right),\;{\text{H}}_{k}={\frac{\partial h}{\partial \text{x}}|}_{{\hat{\text{x}}}_{k|k-1}}$$

The updating phase:

(12d) $${\text{K}}_{k}={\text{P}}_{k|k-1}{\text{H}}_{k}{}^{T}{\left({\text{H}}_{k}{\text{P}}_{k|k-1}{\text{H}}_{k}{}^{T}+{\text{R}}_{k}\right)}^{-1}$$

(12e) $${\tilde{\text{y}}}_{k}={\text{z}}_{k}-{\hat{\text{z}}}_{k}$$

(12f) $${\hat{\text{x}}}_{k|k}={\hat{\text{x}}}_{k|k-1}+{\text{K}}_{k}{\tilde{\text{y}}}_{k}$$

(12g) $${\text{P}}_{k|k}={\text{P}}_{k|k-1}-{\text{K}}_{k}{\text{H}}_{k}{\text{P}}_{k|k-1}$$

where:

**P**(*t_k_*)= **P**_*k*|*k*−1_
**P**(*t*_*k*−1_)= **P**_*k*−1| *k*−1_
**x̄**(*t_k_*)= **x̄**_*k*|*k*−1_
**x̄**(*t_k_*_−1_) = **x̄**_*k*−1| *k*−1_
**F** is the Jacobian of the nonlinear ordinary differential equations
**H** is the Jacobian of the measurement equations
**K** is the gain matrix
**P** is the state error covariance matrix
**x̄** is the estimated state vector
**ỹ** is the measurement residual
**ẑ** is the estimated measurement

Equation 12(b) is a continuous time Riccati equation. In order to solve the equation, the discrete state transition matrix can be determined using Equation 12(h). With the state transition matrix available, Equation 12(i) provides the solution of the state error covariance matrix. Note that Equation 12(h) is applied under the assumption that **F** is a constant from *t*_*k*−1_ to *t*_*k*_:

(12h) $${\text{Φ}}_{k-1}=e^{\text{F}\left(t\right)\Delta t}$$

(12i) $${\text{P}}_{k|k-1}={\text{Φ}}_{k-1}{\text{P}}_{k-1|k-1}{\text{Φ}}_{k-1}^{T}+{\text{Q}}_{k-1}$$

where **Φ** is the state transition matrix

It is important to know that the EKF needs predefined **P**_0|0_, **R**, **Q**, and **x̄**_0|0_ for initialization. It is acceptable to assign **P**_0|0_ arbitrarily, however, the values must be large enough to allow good tracking of the parameters. If the states are measured, **x̄**_0|0_ can be specified by taking the average of the first few data points.

### 5.2. Backward Recursion

The backward recursion works backwards in time. The combination of a forward pass estimator (EKF) and a backward recursion is considered to have utilized all available information [15]. Thus the scheme is capable of performing better estimations than the EKF. Equation 13 summarizes the backward recursion of the RTS smoother:

(13a) $${\text{A}}_{k}={\text{P}}_{k|k}{\text{Φ}}_{k}{}^{T}{\text{P}}_{k+1|k}{}^{-1}$$

(13b) $${\hat{\text{x}}}_{k|n}={\hat{\text{x}}}_{k|k}+{\text{A}}_{k}({\hat{\text{x}}}_{k+1|n}-{\hat{\text{x}}}_{k+1|k}),\;\;k=N-1,\ldots ,0$$

(13c) $${\text{P}}_{k|n}={\text{P}}_{k|k}+{\text{A}}_{k}\left[{\text{P}}_{k+1|n}-{\text{P}}_{k+1|k}\right]{\text{A}}_{k}{}^{T}$$

where:

**A** is the smoother gain matrix
*N* is the final time step
**P**_*k|n*_ is the corresponding state error covariance matrix
**x̄**_*k|n*_ is the smoothed states of *k*^th^ time step

## 6. Flight Tests

### 6.1. The Flight Maneuver

**Figure 5.**
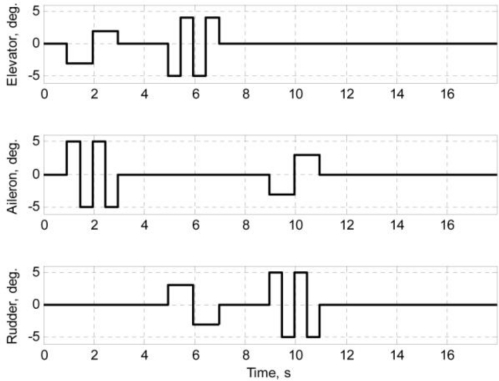
Orthogonal square-wave inputs.

The flight test data were collected through a series of flight tests. In the flight data acquisition process, it is desirable to maneuver the air vehicle such that the flight data contain sufficient information to represent the dynamics of the air vehicle. The maneuvers chosen to fulfill the objective of this work were a multiple-input design. Due to its simplicity, time-skewed doublet inputs like those used in 2008 by Lee *et al.* [7,8] are always a favorable choice, but in order to effectively excite the sufficient dynamic information, including the coupling of the longitudinal and lateral motion, the orthogonal square-wave inputs [17] were applied. The orthogonal square-wave inputs are depicted in Figure 5.

As shown in Figure 5, at any instant, two control surfaces are deflected simultaneously. One is deflected at half the rate of another. The simultaneous deflections are meant to excite couple dynamics, while the separate deflection rates are meant to distinguish the corresponding control surfaces’ effects on the air vehicle motion.

The initial condition of the designed flight maneuver has to be trimmed flight condition. Due to the complexity of the maneuver and the difficulty for the ground pilot to ensure a trimmed flight initial condition, the maneuver was executed by the onboard computer. The ground pilot flew the SP-80 UAV to a desired altitude and remained straight and level flight before switching the control authority to the onboard computer. Once switched, the onboard computer maintained the air vehicle in trimmed straight and level flight for 5 seconds. After that, the designed maneuver was executed and the ground pilot switched it back to manual flight no less than 5 seconds after the maneuver. The desired altitude was about 300 m above ground. Since the air vehicle remained at open-loop control during the maneuver, the 300 m altitude gave the ground pilot enough time to react to any unexpected outcome while the air vehicle remained in the pilot’s eyesight.

It is desirable to perform the flight test under absolutely windless conditions, but this is utterly impossible. Thus, the flight tests were conducted in nearly windless environment and the maneuvers was executed in both head wind and tail wind directions to minimize the crosswind effect.

### 6.2. The Flight Data and Reconstruction

The flight data reconstruction is accomplished by implementing the 4th order Runge-Kutta method. The accelerations and angular rates are the inputs; the airdata, and attitude angles are the outputs. The 4th order Runge-Kutta method is summarized as Equation 14:

(14) $$\begin{array}{l} k_{1}=h\cdot f\left(\text{x}\left(t\right),\text{u}\left(t\right)\right)\\ k_{2}=h\cdot f\left(\left[\text{x}\left(t\right)+\frac{k_{1}}{2}\right],\text{u}\left(t\right)\right)\\ k_{3}=h\cdot f\left(\left[\text{x}\left(t\right)+\frac{k_{2}}{2}\right],\text{u}\left(t\right)\right)\\ k_{4}=h\cdot f\left(\left[\text{x}\left(t\right)+k_{3}\right],\text{u}\left(t\right)\right)\\ \text{x}\left(t+h\right)=\text{x}\left(t\right)+\frac{k_{1}+2k_{2}+2k_{3}+k_{4}}{6} \end{array}$$

where *h* is the time step size

**Figure 6.**
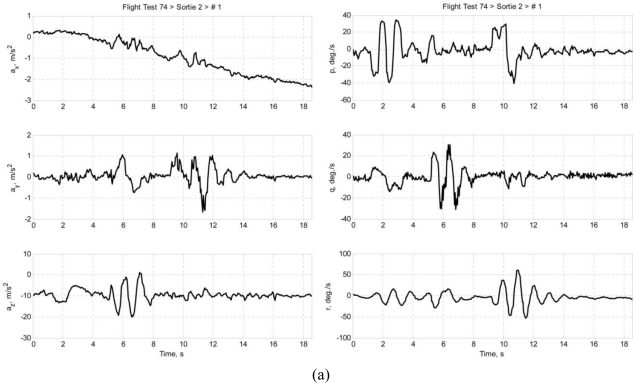

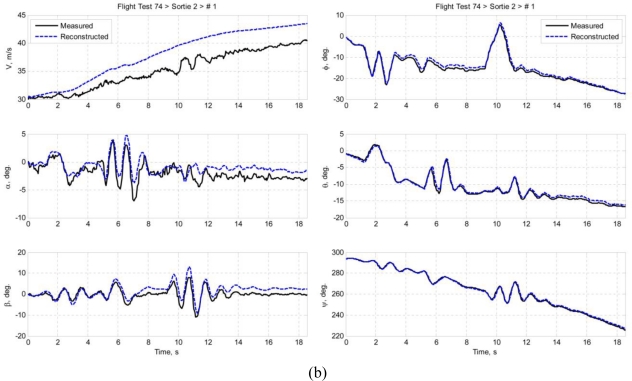
**(a)** Accelerations and angular rates of *ft74srt02run01*. **(b)** Airdata and attitude angles of *ft74srt02run01*.

**Figure 7.**
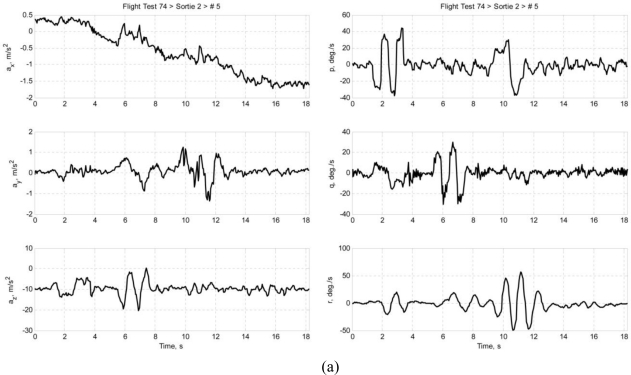

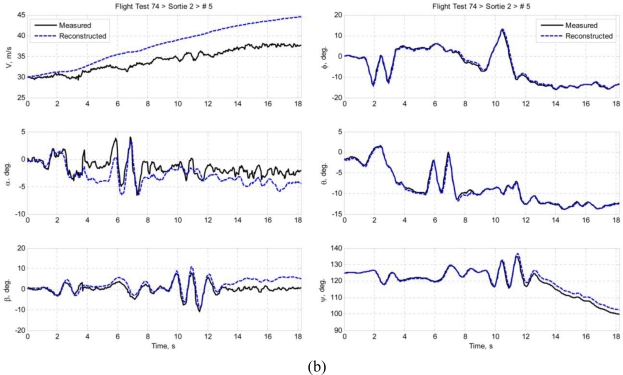
**(a)** Accelerations and angular rates of *ft74srt02run05*. **(b)** Airdata and attitude angles of *ft74srt02run05*.

Multiple sets of flight data were collected and each data set is designated in this paper in the following format: *ft*FF*srt*SS*run*RR, where FF is the flight test number of the Spoonbill UAV project, SS is the sortie number, and RR is the number of maneuver in that particular sortie. This paper presents two data sets as examples: *ft74srt02run01* and *ft74srt02run05*. Data of *ft74srt02run01* is depicted in Figure 6, and Figure 7 corresponds to *ft74srt02run05*. Both data sets show similar trends. The reconstructed attitude angles demonstrate very good compatibility while the airdata compatibility is poor.

From Equation 7, it is legitimate to infer that the airspeed, AoA, and AoS variations are dominated by X-axis acceleration, Z-axis acceleration, and Y-axis acceleration, respectively. The reconstructed airspeed is over estimated in both data sets. It is obvious that the reconstructed airspeeds are increasing at a higher rate compared to the measured airspeed, which suggest the presence of a bias on the X-axis acceleration. Fluctuations are observed on the measured airspeeds at the 9th to 12th second, but not on the reconstructed airspeeds. That could be the result of instrumentation errors of the airdata probe due to rapid yawing motion of the air vehicle. The AoA and AoS demonstrate slightly better compatibilities compared to the airspeed. The reconstructed data seem to follow the trend of the measured data. This suggests that the biases of Y-axis acceleration and Z-axis acceleration are less significant. Thus, the instrumentation error, measurement noises, and atmospheric turbulent are the major causes of the AoA and AoS incompatibilities.

Similar inferences were made on Equation 8. The roll angle, pitch angle, and yaw angle variations are dominated by the roll rate, pitch rate, and yaw rate, respectively. It is well understood that the attitude angles are estimated through complex algorithms using the angular rates, and accelerations [18]. The principle idea is that the attitude angles are estimated through integration of the angular rates, and the accelerations prevent the drifting of the estimation. That explains the high compatibilities of the attitude angles. Since the attitude angles estimation is augmented by GPS data [19,20], it is more likely that the attitude angles are relatively accurate and noises of the angular rates are the reason of the slight incompatibilities.

## 7. Sensor Data Compatibility Correction

### 7.1. The Compatibility Correction Procedure

**Figure 8.**
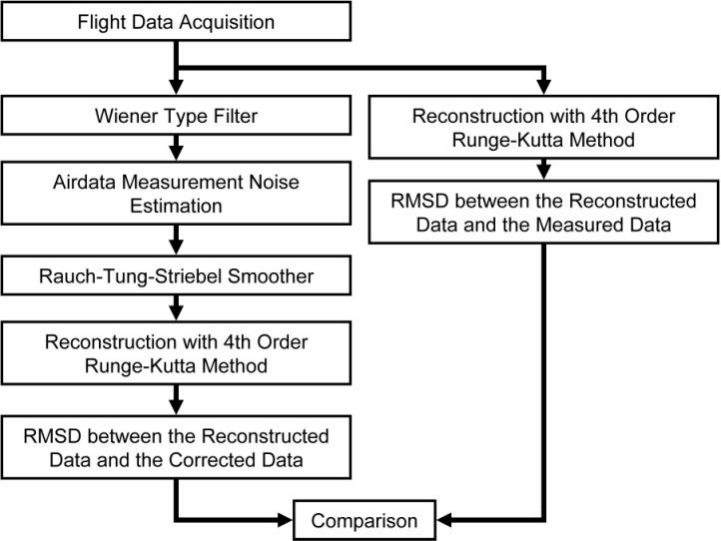
The compatibility correction procedure.

The sensor compatibility correction procedure as illustrated in Figure 8 was designed under several assumptions:

- The AHRS body axes and the air vehicle body axes coincide.
- The airdata probe is located near to the center of gravity such that position correction is unnecessary.
- Since the AHRS is not exposed to the environment, the AHRS readings (accelerations, angular rates, and attitude angles) are less affected by atmospheric turbulence, and the noises (not the biases) are high frequency noises that can be effectively filtered by a frequency filter.
- Due to the high compatibility of the attitude angles, the biases of the angular rates are negligible.
- The noises or errors of different variables are not inter-correlated.
- The noises distributions are Gaussian.

As seen in the right portion of Figure 8, the flight data undergo reconstruction using the 4th order Runge-Kutta Method. The reconstructed results of *ft74srt02run01* and *ft74srt02run05* are presented in the previous section as Figure 6 and Figure 7. The RMSD shown here as Equation 15 is then implemented to quantify the quality of compatibility. The smaller the RMSD is, the better the compatibility is.

The RMSD between the measured and reconstructed airdata and attitude angles are calculated. This is the RMSD before the correction:

(15) $$\mathrm{RMSD}\left({\text{γ}}_{1},{\text{γ}}_{2}\right)=\sqrt{\frac{\sum_{i=1}^{n_{d}}{\left(\gamma_{1,i}-\gamma_{2,i}\right)}^{2}}{n_{d}}}$$

where:

*n_d_* is the data size
**γ**_1_ is the first data
**γ**_2_ is the second data
**γ**_1,*i*_ is the i^th^ element of the first data
**γ**_2,*i*_ is the i^th^ element of the second data

The left portion of Figure 8 illustrates the correction process. The RTS smoother is the core of the whole process. The flight data (airspeed, AoA, AoS, attitude angles, angular rates, and accelerations) is first passed through a Wiener Type Filter to suppress the high frequency noises of the flight data. It then followed by the airdata measurement noise estimation that will be discussed in depth in Section 7.3. RTS is implemented after that to correct the flight data such that the data is compatible to the force equations and kinematic equations. The corrected accelerations and angular rates are then used as inputs to perform airdata and attitude angle reconstruction. If successful, the corrected and the reconstructed airdata and attitude angles should show a high degree of similarity. After that, the RSMD between the corrected and the reconstructed outputs (airdata and attitude angles) are calculated. This is the RMSD after the correction. Finally the RMSDs before and after the correction are compared to demonstrate the sensor data compatibility improvement. For the work presented in this paper, the RTS was executed with 0.05 s sampling time, which is consistent with the 20 Hz sampling rate of the onboard system.

### 7.2. RTS Problem Formulation

Under the assumptions discussed in the previous section, the RTS problem formulation is as shown in Equations 16–18. Equation 16 shows the relation between the measured accelerations and the corresponding biases. As in Equation 17, the biases are formulated as the state variables such that the RTS smoother could estimate them. The airdata and the attitude angles are part of the state variables and they are also the measurement. The accelerations and angular rates are the inputs. While the force equations and kinematic equations serve as the ordinary differential equations of corresponding states, the acceleration biases are assumed constant as shown in Equation 18:

(16a) $$a_{x_{M}}=a_{x}+b_{a_{x}}$$

(16b) $$a_{y_{M}}=a_{y}+b_{a_{y}}$$

(16c) $$a_{z_{M}}=a_{z}+b_{a_{z}}$$

where:

*a*_*x*_*M*__ is the measured X-axis acceleration
*a*_*y*_*M*__ is the measured Y-axis acceleration
*a*_*z*_*M*__ is the measured Z-axis acceleration
*b*_*a*_*x*__ is the X-axis acceleration measurement bias
*b*_*a*_*y*__ is the Y-axis acceleration measurement bias
*b*_*a*_*z*__ is the Z-axis acceleration measurement bias

(17a) $$\text{x}={\left[V\;\;\alpha \;\;\beta \;\;\phi \;\;\psi \;\;\text{b}\right]}^{T},\text{b}={\left[b_{a_{x}}\;\;b_{a_{y}}\;\;b_{a_{z}}\right]}^{T}$$

(17b) $$\text{z}={\left[V\;\;\alpha \;\;\beta \;\;\phi \;\;\theta \;\;\psi \right]}^{T}$$

(17c) $$\text{u}={\left[a_{x_{M}}\;\;a_{y_{M}}\;\;a_{z_{M}}\;\;p\;\;q\;\;r\right]}^{T}$$

where **b** is the acceleration measurement bias vector:

(18) $$\dot{\text{b}}=0_{3\times 1}$$

### 7.3. Measurement Noise & Process Noise Covariance Matrix Estimation

It was assumed that the noises are not inter-correlated in order to simplify the noise covariance matrices estimation. Hence, the task is to estimate the diagonal terms of the matrices, namely the variances. The measurement noise variances of the airdata are estimated from the speeds measured by the GPS. Unlike the airdata, the GPS speed measurements are less noisy. That is because the GPS doesn’t measure speed by interacting with the relative airflow like the airdata probe does. In addition, the atmospheric turbulence is the major contributor to the airdata noise or error. One might argue that it is impossible to accurately estimate the airdata measurement noise variances from the GPS speed measurements because the airdata are relative to the wind, but the GPS measurements are relative to the ground. But it is later demonstrated that the GPS speed measurements are sufficient to fulfill the purpose.

The GPS speed measurement would have to go through a series of coordinate transformations before any noise variances estimation is possible. Equation 19 transforms the horizontal ground speed and ascent rate measured by the GPS to the North-East-Down (NED) local horizontal coordinate frame. The NED speed components are then transformed to the air vehicle body-axis using Equation 20. After that, the body-axis speeds are substituted into Equation 21 to obtain estimations of total speed, AoA, and AoS. It is essential to take note that these estimations are not meant to be accurate, but since they are far much less sensitive to atmospheric turbulent, these estimations provide a good baseline reference for the airdata measurement noise variances estimation:

(19a) $$V_{N,\mathrm{gps}}=V_{\mathrm{hor},\mathrm{gps}}\;\text{cos}\;\psi$$

(19b) $$V_{E,\mathrm{gps}}=V_{\mathrm{hor},\mathrm{gps}}\;\text{sin}\;\psi$$

(19c) $$V_{D,\mathrm{gps}}=-V_{U,\mathrm{gps}}$$

where:

*V_hor,gps_* is the horizontal speed given by GPS
*V_E,gps_* is the ground speed to the East
*V_N,gps_* is the ground speed to the North
*V_U,gps_* is the ascent rate given by GPS
*V_D,gps_* is the descent rate

(20) $$\left[\begin{array}{c} u_{\mathrm{gps}}\\ v_{\mathrm{gps}}\\ w_{\mathrm{gps}} \end{array}\right]=\left[\begin{array}{ccc} 1 & 0 & 0\\ 0 & \text{cos}\;\phi & \text{sin}\;\phi \\ 0 & -\text{sin}\;\phi & \text{cos}\;\phi \end{array}\right]\left[\begin{array}{ccc} \text{cos}\;\theta & 0 & -\text{sin}\;\theta \\ 0 & 1 & 0\\ \text{sin}\;\theta & 0 & \text{cos}\;\theta \end{array}\right]\left[\begin{array}{ccc} \text{cos}\;\psi & \text{sin}\;\psi & 0\\ -\text{sin}\;\psi & \text{cos}\;\psi & 0\\ 0 & 0 & 1 \end{array}\right]\left[\begin{array}{c} V_{N,\mathrm{gps}}\\ V_{E,\mathrm{gps}}\\ V_{D,\mathrm{gps}} \end{array}\right]$$

where:

*u_gps_* is the body X-axis speed estimated from GPS measured speeds
*v_gps_* is the body Y-axis speed estimated from GPS measured speeds
*w_gps_* is the body Z-axis speed estimated from GPS measured speeds

(21a) $$V_{\mathrm{gps}}=\sqrt{u_{\mathrm{gps}}{}^{2}+v_{\mathrm{gps}}{}^{2}+w_{\mathrm{gps}}{}^{2}}$$

(21b) $$\alpha_{\mathrm{gps}}={\text{tan}}^{-1}\left(\frac{w_{\mathrm{gps}}}{u_{\mathrm{gps}}}\right)$$

(21c) $$\beta_{\mathrm{gps}}={\text{tan}}^{-1}\left(\frac{v_{\mathrm{gps}}}{V_{\mathrm{gps}}}\right)$$

where:

*V_gps_* is the GPS total speed
*α_gps_* is the GPS estimated AoA
*β_gps_* is the GPS estimated AoS

The airdata estimations given by Equation 21 serve as reference for the airdata measurement noise estimations. The most intuitive way of estimating the noise is determining the residual between the airdata estimations with the airdata measurements. However, the airdata estimations come with inherent biases due to the difference between airspeed and ground speed. Instead, the measurement noise estimations are derived based on the deviation of the airdata at any particular time with their local expected values. The idea is as shown in Equation 22. Take Equation 22(a) as an example, the first portion of the equation tells how far is the *k*^th^ airspeed measurement deviate from its local expected value. The local expected value is the mean of airspeed measurements from (*k* − *m*)^th^ to (*k* + *m*)^th^ time steps. Similarly, in the second portion of the equation, the deviation of the *k*^th^ GPS total speed from its expected value is calculated. The difference between these two deviations gives the estimated noise of the *k*^th^ airspeed measurement. One can control the local expected values by changing *m*. If the air vehicle is highly dynamic, a smaller *m* gives more legitimate local expected values:

(22a) $${ɛ}_{V,k}=\left[V_{k}-\frac{1}{2m+1}\sum_{i=k-m}^{2m+1}V_{i}\right]-\left[V_{\mathrm{gps},k}-\frac{1}{2m+1}\sum_{i=k-m}^{2m+1}V_{\mathrm{gps},i}\right]$$

(22b) $${ɛ}_{\alpha ,k}=\left[\alpha_{k}-\frac{1}{2m+1}\sum_{i=k-m}^{2m+1}\alpha_{i}\right]-\left[\alpha_{\mathrm{gps},k}-\frac{1}{2m+1}\sum_{i=k-m}^{2m+1}\alpha_{\mathrm{gps},i}\right]$$

(22c) $${ɛ}_{\beta ,k}=\left[\beta_{k}-\frac{1}{2m+1}\sum_{i=k-m}^{2m+1}\beta_{i}\right]-\left[\beta_{\mathrm{gps},k}-\frac{1}{2m+1}\sum_{i=k-m}^{2m+1}\beta_{\mathrm{gps},i}\right]$$

where:

*ɛ* is the estimated noise/error
*m* is the design parameter

**Figure 9.**
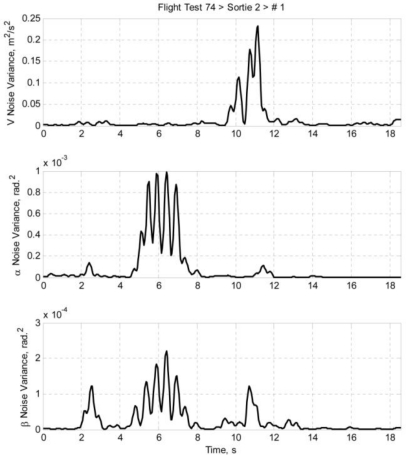
Airdata noise variance of *ft74srt02run01*.

**Figure 10.**
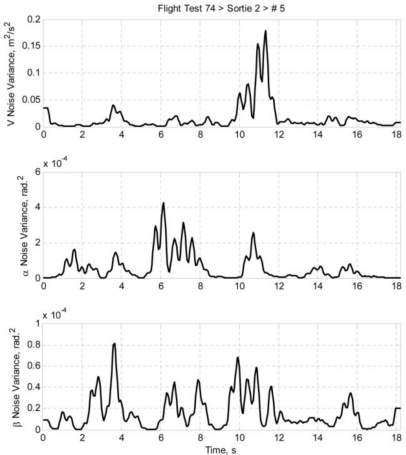
Airdata noise variance of *ft74srt02run05*.

Once the airdata measurement noises are estimated using Equation 22, the noise variances can be calculated. Using the same idea of data regionality, the noise variances are calculated by taking the nearby data points into account. The equations are presented as Equation 23. Taking Equation 23(a) as an example, it is a standard variance equation; the *k*^th^ airspeed measurement noise variance is calculated by accounting a sample with sample size of (2 *m* + 1). The larger the *m* is, the larger the sample size is, and thus the less intense the variation of the noise variance is. Figures 9 and 10 depict the estimated airdata measurement noise variance of *ft74srt02run01* and *ft74srt02run05* at *m* = 5:

(23a) $$\sigma_{{ɛ}_{V},k}^{2}=\frac{1}{2m+1}{\sum_{i=k-m}^{2m+1}\left({ɛ}_{V,i}-{\bar{ɛ}}_{V}\right)}^{2},\;{\bar{ɛ}}_{V}=\frac{1}{2m+1}\sum_{i=k-m}^{2m+1}\left({ɛ}_{V,i}\right)$$

(23b) $$\sigma_{{ɛ}_{\alpha },k}^{2}=\frac{1}{2m+1}{\sum_{i=k-m}^{2m+1}\left({ɛ}_{\alpha ,i}-{\bar{ɛ}}_{\alpha }\right)}^{2},\;{\bar{ɛ}}_{\alpha }=\frac{1}{2m+1}\sum_{i=k-m}^{2m+1}\left({ɛ}_{\alpha ,i}\right)$$

(23c) $$\sigma_{{ɛ}_{\beta },k}^{2}=\frac{1}{2m+1}{\sum_{i=k-m}^{2m+1}\left({ɛ}_{\beta ,i}-{\bar{ɛ}}_{\beta }\right)}^{2},\;{\bar{ɛ}}_{\beta }=\frac{1}{2m+1}\sum_{i=k-m}^{2m+1}\left({ɛ}_{\beta ,i}\right)$$

where:

$\sigma_{{ɛ}_{V}}^{2}$ is the estimated error variance of airspeed
$\sigma_{{ɛ}_{\alpha }}^{2}$ is the estimated error variance of AoA
$\sigma_{{ɛ}_{\beta }}^{2}$ is the estimated error variance of AoS

Under the assumptions given in Section 7.1, the measurement noise covariance is a diagonal matrix and the attitude angles measurement noise variances are zeros after the implementation of the Wiener Type Filter. Hence, the measurement noise covariance matrix is in the following form:

(24) $${\text{R}}_{k}=\left[\begin{array}{cc} \begin{array}{ccc} \sigma_{{ɛ}_{V},k}^{2} & 0 & 0\\ 0 & \sigma_{{ɛ}_{\alpha },k}^{2} & 0\\ 0 & 0 & \sigma_{{ɛ}_{\beta },k}^{2} \end{array} & 0_{3,3}\\ 0_{3,3} & 0_{3,3} \end{array}\right]$$

On the other hand, the process noise is mainly contributed by the accelerations and the angular rates. If the second assumption in Section 7.1 is valid, the process noise covariance matrix shall be a zero matrix since the data has already been filtered by the Wiener Type Filter. However, a zero process noise covariance matrix will lead the RTS smoother to a singularity. Thus, the process noise covariance matrix was chosen to be a diagonal matrix with very small diagonal terms as shown in Equation 25:

(25) $${\text{Q}}_{k}•\text{I}\times 10^{-5}$$

## 8. Results and Discussion

**Figure 11.**
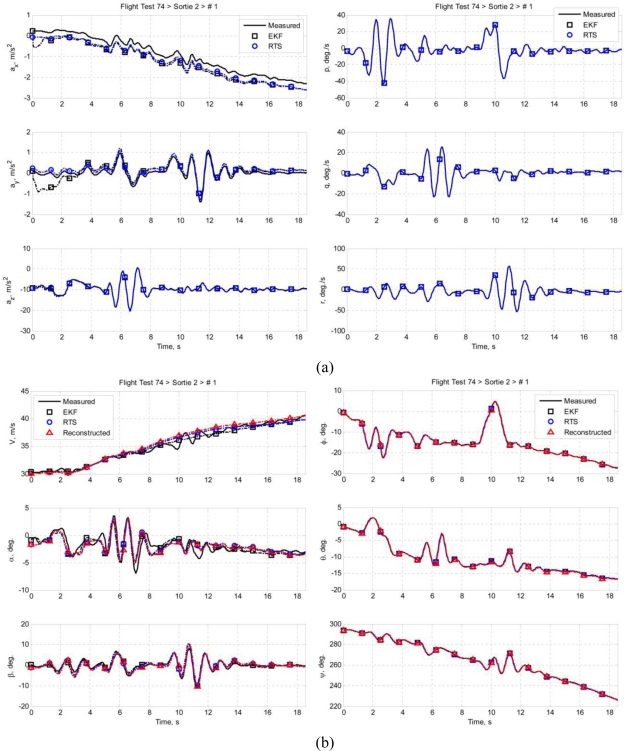
**(a)** Accelerations and angular rates of *ft74srt02run01* before and after correction. **(b)** Airdata and attitude angles of *ft74srt02run01* before and after correction.

**Figure 12.**
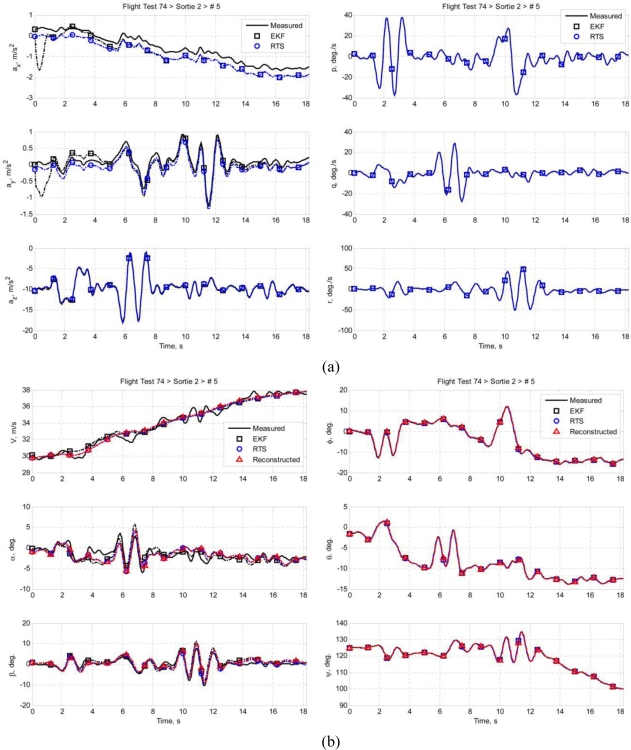
**(a)** Accelerations and angular rates of *ft74srt02run05* before and after correction. **(b)** Airdata and attitude angles of *ft74srt02run05* before and after correction.

Figures 11 and 12 present the results of the sensor compatibility correction of *ft74srt02run01* and *ft74srt02run05*, respectively. The figures contain correction results using the EKF (the RTS forward pass), the RTS (the RTS forward pass and backward recursion), and the measured data (after Wiener Type Filtering at cutoff frequency of 2 Hz). As shown in Figure 11(a) and Figure 12(a), the EKF took some time before the acceleration biases converged, and the biases remain constant in the backward recursion. This is due to the inherent characteristics of the RTS backward recursion whereby it is unable to update the estimations with zero change rates. However, the biases are meant to be constants, and as long as the EKF provide converged estimations, the biases are valid and the RTS backward recursion helps applying the converged biases to all data points.

High degree of similarities is demonstrated on the angular rates and attitude angles. Comparing Figure 6 and Figure 11, and Figure 7 and Figure 12, the compatibilities of the attitude angles improved slightly. The EKF and RTS have done little correction on the attitude angles; the result is mainly due to the high frequency noises suppression by the Wiener Type Filter. This is coherent with the assumption that the AHRS measurement contains only high frequency noises that can be effectively filtered by a frequency filter, and the biases of the angular rates are negligible. Also demonstrated is the significant improvement of the airdata compatibility. The reconstructed airspeed tracks the corrected airspeed with good consistency. The AoA and AoS also show significant improvement. One of the major reasons of the significant improvement of the airspeed compatibility is that the RTS has successfully identified a significant bias on the X-axis acceleration as suggested in Section 6.2. The improvements on the AoA and AoS compatibilities are due to the noise reduction on the angular rates and the biases estimation on the corresponding accelerations.

Tables 1 and 2 demonstrate the quantitative improvement by comparing the RMSDs before and after correction. The compatibilities of all the airdata and attitude angles have improved. The airspeed has the best improvement with RMSD reduction of as high as 97.76%, which is consistent with the significant X-axis acceleration bias estimated in both *ft74srt02run01* and *ft74srt02run05*. Although the improvements of the attitude angles compatibilities are not apparent in Figures 11 and 12, all of the attitude angles have shown at least 47.43% of RMSD reduction.

## 9. Conclusions

This paper presented an easy and straightforward procedure for implementing sensor compatibility corrections for fixed-wing UAVs. The procedure implemented the Wiener Type Filter, the RTS Smoother, and an airdata noise variances estimation method. The Wiener Type Filter was introduced to filter out the high frequency noises of the flight data, thus simplifying the measurement noise matrix and process noise matrix estimation. The RTS Smoother serves as the main correction algorithm. It is a two pass algorithm that combines the EKF and a backward recursion. The airdata noise variances estimation method accomplishes its purpose using the GPS speed measurements.

The sensor data compatibility correction procedure has been successfully implemented on real flight data recorded on the SP-80 UAV of the RMRL. Initial data reconstruction shows that the compatibility is poor and the airdata is far much noisier than the AHRS data due to the exposure of the airdata probe to the atmospheric turbulence. It was assumed that the AHRS data noises only consist of high frequency noises and the noises can be effectively filtered by the Wiener Type Filter. Also, due to the high compatibility of the attitude angles, it was assumed that the biases of the angular rates are negligible. Since the airdata is corrupted by atmospheric turbulence, the Wiener Type Filter was unable to suppress all the noises. Thus, relatively accurate airdata noise variances are needed. The airdata noise variances estimation method brings about the concept of data regionality. Under this concept, the data around a particular data point are assumed to have statistical relation with the data point. This has allowed the estimation of the expected value and variance at any particular data point by taking the data around the data point into account. This concept might not apply universally to all problems, but it has adequately fulfilled its purpose for the work presented in this paper.

After the correction, significant sensor compatibility improvement, especially on the airspeed data has been demonstrated. The improvement of the airdata compatibilities is mainly contributed by the estimation of the acceleration biases by the RTS. On the other hand, the Wiener Type Filter contributes to the improvement of attitude angle compatibilities.

The main benefit of the sensor data compatibility correction is to provide better data reliability, which leads to a better understanding of the dynamics of an air vehicle. Such a correction procedure is essential if an accurate dynamic model is to be identified using the flight data. The Wiener Type Filter and RTS are both batch (offline) methods. Future improvement to the procedure could include the implementation of a real-time (online) algorithm. Being able to perform sensor compatibility correction in real-time could help improve the robustness of the autopilot system for both manned and unmanned vehicles.

## Figures and Tables

**Table 1.** Comparison of RMSD before and after correction of *ft74srt02run01*.

	
	**Root Mean Square Deviation (RMSD)**		**RMSD Reduction, %**
	**Before Correction**	**After Correction**	
**Airspeed (***V***), m/s**	2.9855	0.3373	88.70
**AoA (***α***), deg.**	1.3855	0.3490	74.81
**AoS (***β***), deg.**	2.3143	0.7520	67.51
**Roll Angle (***ϕ***), deg.**	1.0314	0.3086	70.08
**Pitch Angle (***θ***), deg.**	0.5413	0.2121	60.82
**Yaw Angle (***ψ***), deg.**	0.8486	0.4190	50.62

**Table 2.** Comparison of RMSD before and after correction of *ft74srt02run05*.

	
	**Root Mean Square Deviation (RMSD)**		**RMSD Reduction, %**
	**Before Correction**	**After Correction**	
**Airspeed (***V***), m/s**	3.3453	0.0751	97.76
**AoA (***α***), deg.**	1.7380	0.4319	75.15
**AoS (***β***), deg.**	2.6914	0.9741	63.81
**Roll Angle (***ϕ***), deg.**	0.7183	0.3625	49.53
**Pitch Angle (***θ***), deg.**	0.4090	0.2138	47.73
**Yaw Angle (***ψ***), deg.**	1.6547	0.3729	77.46
